# The significance of leptomeningeal collaterals in moyamoya disease

**DOI:** 10.1111/cns.13389

**Published:** 2020-05-01

**Authors:** Jin Yu, Jibo Zhang, Jincao Chen

**Affiliations:** ^1^ Department of Neurosurgery Zhongnan Hospital of Wuhan University Wuhan China

Dear Editor,

We read with great interest the article by Liu et al^1^ regarding the clinical characteristics and leptomeningeal collateral (LMC) status in pediatric and adult patients with ischemic moyamoya disease (MMD). The authors observed that pediatric MMD patients have greater patency and a greater ability to establish good LMC status than adult patients, and poor LMC status has a strong correlation with severe clinical symptoms and poor postoperative outcomes. We really appreciate the interesting observation for their conclusion. Meanwhile, after reading this article, we would like to highlight 2 important questions that it raises.

First, the formation of LMC is believed to compensate for intracranial ischemia. The authors found that pediatric MMD owns more effective LMC than adults MMD, which means that pediatric MMD have more compensatory vascular anastomosis responded to the low perfusion of the ischemic area. Indeed, according to previous studies, ischemic MMD was more common in pediatric patients than in adult patients. ^2^ However, the anastomosis of PCA and ACA/ MCA will also increase the hemodynamic burden of posterior circulation and increase the risk of hemorrhage.^3^ This should be noted during the clinical management of pediatric MMD patients. Meanwhile, other compensatory collaterals, such as extracranial arterial collateral circulation anastomoses from the facial, maxillary and middle meningeal arteries to the ophthalmic artery, and dural arteriolar anastomoses from the middle meningeal artery and occipital artery through the mastoid foramen and parietal foramen, may also have correlation with postoperative outcomes.^4^

Second, the aim of extra‐intra cranial bypass surgery is to guide extracranial blood flow into the intracranial ischemic area through the recipient vessel to improve perfusion. Therefore, the supply area and hemodynamic source of the recipient vessel are very important for the postoperative effect of bypass surgery. According to the authors, the wide range of complex LMC in the brain of MMD patients should lead to a variety of hemodynamic sources for parasylvian continental arteries (PSCAs). Indeed, in our latest observations,^5^ we observed different hemodynamic sources of the recipient PSCAs among the frontal, temporal, and parietal PSCAs in MMD hemispheres. These results suggested that the recipient vessel in traditional “superficial temporal artery‐middle cerebral artery (STA‐MCA)” bypass surgery was not always from MCA. Therefore, neurosurgeons should rely more on digital subtraction angiography to determine the hemodynamic source of recipient vessels than on anatomy.

## CONFLICT OF INTEREST

We certify that we have no affiliations with any organization or entity with any financial interest or non‐financial interest.

## FUNDING INFORMATION

This research did not receive any specific grant from funding agencies in the public, commercial, or not‐for‐profit sectors.
